# Nuclear Deformation in Response to Mechanical Confinement is Cell Type Dependent

**DOI:** 10.3390/cells8050427

**Published:** 2019-05-08

**Authors:** Mary T. Doolin, Thea S. Ornstein, Kimberly M. Stroka

**Affiliations:** 1 Fischell Department of Bioengineering, University of Maryland, College Park, MD 20742, USA; mdoolin@terpmail.umd.edu (M.T.D.); theao17@gmail.com (T.S.O.); 2 Biophysics Program, University of Maryland, College Park, MD 20742, USA; 3 Center for Stem Cell Biology and Regenerative Medicine, University of Maryland, Baltimore, MD 21201, USA; 4 Marlene and Stewart Greenebaum Comprehensive Cancer Center, University of Maryland, Baltimore, MD 21201, USA

**Keywords:** nucleus, confinement, mesenchymal stem cell, fibroblast

## Abstract

Mechanosensing of the mechanical microenvironment by cells regulates cell phenotype and function. The nucleus is critical in mechanosensing, as it transmits external forces from the cellular microenvironment to the nuclear envelope housing chromatin. This study aims to elucidate how mechanical confinement affects nuclear deformation within several cell types, and to determine the role of cytoskeletal elements in controlling nuclear deformation. Human cancer cells (MDA-MB-231), human mesenchymal stem cells (MSCs), and mouse fibroblasts (L929) were seeded within polydimethylsiloxane (PDMS) microfluidic devices containing microchannels of varying cross-sectional areas, and nuclear morphology and volume were quantified via image processing of fluorescent cell nuclei. We found that the nuclear major axis length remained fairly constant with increasing confinement in MSCs and MDA-MB-231 cells, but increased with increasing confinement in L929 cells. Nuclear volume of L929 cells and MSCs decreased in the most confining channels. However, L929 nuclei were much more isotropic in unconfined channels than MSC nuclei. When microtubule polymerization or myosin II contractility was inhibited, nuclear deformation was altered only in MSCs in wide channels. This work informs our understanding of nuclear mechanics in physiologically relevant spaces, and suggests diverging roles of the cytoskeleton in regulating nuclear deformation in different cell types.

## 1. Introduction

The way a cell senses and responds to a physical force can vary based on cell type or tissue microenvironment, potentially altering downstream events such as migration, homeostasis, differentiation, proliferation, or tumorigenesis [1,2]. An important element in the force transmission pathway is the nucleus, the largest and stiffest cell organelle [3]. The nucleus has been characterized as a mechanosensor of the cell and transmits external forces emanating from the cellular microenvironment to the nuclear envelope housing the chromatin [4,5,6]. Mechanical forces propagate from the extracellular matrix (ECM) to mechanosensitive focal adhesions and cytoskeletal structures. Cytoskeletal structures are then connected to the nuclear lamina, below the nuclear envelope, through the linker of nucleoskeleton and cytoskeleton (LINC) complex [5]. Additionally, direct force application to the nucleus can induce chromatin stretching and transcriptional upregulation of a reporter transgene without involvement of the nucleo-cytoskeleton network [7]. Isolated and intracellular nuclei can deform anisotropically in response to both active and passive externally applied forces. Active forces include atomic force microscopy (AFM) [8], biaxial stretching devices [9], and magnetic tweezers [10], while passive forces include glass microtubes [11], three-dimensional (3D) hydrogels [12], and microfluidic devices [13,14,15,16,17]. Using AFM and micropipette nuclear aspiration, the nucleus can physically deform with as little as a few nanonewtons of force [8,18]. Specifically, differentiated and undifferentiated nuclei alike preferentially deform along their minor (short) axis in reaction to an applied force [8,9,19]. We have previously shown that the nuclear major (long) axis in human mesenchymal stem cells (MSCs) maintains a constant length with increasing degrees of lateral confinement [13]. In contrast, we have also shown that nuclear major (long) axis in mouse sarcoma (Ab3) cells increases in length with increasing degrees of lateral confinement [14]. While anisotropic deformation has been found to be inherent to nuclei of many different cell types, nuclear axis lengths in three dimensions have not been quantified in confinement.

Physical confinement is experienced by cells in vivo in many contexts. For example, cells may be confined within channels between connective tissue and the basement membrane of muscle, nerve, or epithelium [20,21], during cell intravasation or extravasation [22], in a tumor microenvironment [23], or in interstitial tissue [24]. Indeed, the migration modes of several cell types are altered by the degree of confinement induced [20,24,25,26,27]. Although a vast array of cell types experience mechanical confinement, the components involved in mechanotransduction can differ among cell types. Cytoskeleton prestress, the composition of the LINC complex, and the organization of the nucleoskeleton can differ based on cell type [10]. Specifically, increased expression of lamin A has been shown to correlate with increased nuclear, cell, and tissue stiffness [28]. Furthermore, the sensitivity of a particular cell type to nuclear anisotropy is influenced by lamin A expression as well as chromatin organization, and lamin A over-expression results in an increase in strain along the nuclear minor axis in response to an applied force [8]. Additionally, the deformation of the nuclear major and minor axes can be influenced by microtubule and actin structures. In short-term biaxial stretching experiments with epithelial monolayers, it was discovered that microtubules resist deformation along the major axis, while actin resists deformation along the minor axis [9]. Microtubule and actin networks have also been known to regulate cell volume, which can dynamically alter nuclear morphology [29]. In micro-engineered 3D environments, the ratio of cellular to nuclear volume is conserved, but the specific volumes of the nucleus and cell are dependent on cell type [30]. It remains unknown whether cytoskeletal proteins influence nuclear deformability and nuclear volume in confined migration, and whether their role differs in varying cell types.

In this work, we characterized the 3D morphology of nuclei within two cell types in response to physical confinement. Cell types were chosen for experimentation due to their differences in tissue origin, organism origin, disease pathology, and level of differentiation potency. To recapitulate and control the degree of confinement experienced by cells in vitro, we employed polydimethylsiloxane (PDMS)-based microchannels that were 30, 60, 100, 200, or 500 μm^2^ in cross-sectional area, which was previously designed and characterized [16,17]. Nuclear deformation in the XYZ planes, as well as nuclear area and volume, were quantified using immunofluorescence and ImageJ software. The effects of myosin II inhibition and microtubule depolymerization on nuclear deformation were additionally investigated. Our results suggest that cell type-dependent phenotypes may possess a larger role in governing nuclear deformation than the force of confinement alone, and that actin and microtubules may not independently modulate nuclear anisotropy in confined cell migration.

## 2. Materials and Methods

### 2.1. Cell Culture and Reagents

Bone marrow-derived human MSCs (donor: 20-year-old female) were purchased (RoosterBio Inc., Frederick, MD, USA) and experimentally used up until passage 5, which is when the population reaches a doubling level of 20. Human breast adenocarcinoma highly metastatic cells (MDA-MB-231 cells; American Type Culture Collection, Manassas, VA, USA) were used up to a passage of 30 after purchase. Finally, adult mouse fibroblasts (L929 cells) were gifted from Dr. John Fisher (Fischell Department of Bioengineering at the University of Maryland, College Park, MD, USA) and were used up to a passage of 10.

All cell types were cultured in either T-25 or T-75 polystyrene, plasma-treated flasks (VWR, Radnor, PA) and grown in medium comprised of 89% Dulbecco’s Modified Eagle’s Medium with high glucose (DMEM; ThermoFisher Scientific, Waltham, MA, USA), 10% heat-inactivated fetal bovine serum (FBS; ThermoFisher Scientific, Waltham, MA, USA), and 1% penicillin/streptomycin 1000 U/mL (P/S; ThermoFisher Scientific, Waltham, MA, USA). All cells were cultured at 37 °C, with 50% humidity and 5% CO_2_:95% air, and passaged at or below 90% confluency. Cells to be passaged were first washed in phosphate buffered saline (PBS; VWR, Radnor, PA) and detached from the flasks with 0.25% Trypsin-EDTA (ThermoFisher Scientific, Waltham, MA, USA), except for MSCs, which were treated with TrypLE Express Enzyme (ThermoFisher Scientific, Waltham, MA, USA).

### 2.2. Microfluidic Device Manufacturing and Cell Seeding

Polydimethylsiloxane (PDMS) microfluidic devices were prepared according to previously described protocols [13,16,17]. The devices contained either a variety of channel widths or a repeating, single channel width. All photolithography procedures were carried out in the University of Maryland Nanocenter FabLab. In summary, separate masks were designed in AutoCAD (AutoDesk, San Rafael, CA, USA) for the channels (first feature) and the seeding and collection reservoirs (second feature). A 4-inch diameter silicon wafer (University Wafer, Boston, MA, USA) was spin coated with a 10-μm thick layer of SU-8-3010 negative photoresist (MicroChem, Westborough, MA, USA), and the mask representing the channels was placed over the coated wafer using the EVG620 mask aligner (EVG Group, Albany, NY, USA). The layers were then selectively exposed to UV to complete crosslinking of the SU-8-3010 in the areas dictated by the mask. Remaining SU-8-3010 was then dissolved using SU-8 developer (MicroChem). To create the second feature, a layer of SU-8 3025 negative photoresist (MicroChem, Westborough, MA, USA) was spin coated onto the wafer. Then, the EVG620 mask aligner was used to place the mask of the seeding and collection reservoirs over the coated wafer. The system was once again selectively exposed to UV, and the extra SU-8-3025 was removed. The completed wafers were silanized using 97% tridecafluoro-1,1,2,2, tetrahydrooctyl-1-trichlorosilane (UCT Inc., Bristol, PA, USA) overnight in a vacuum desiccator. The finished silicon masters contained the mold for manufacturing single- or multi-microchannel devices, defined by the widths of their channels, which included 50, 20, 10, 6, and 3 μm. Regardless of channel width, all channels were 10 μm in height and 200 μm in length.

PDMS devices were fabricated from the silicon masters. PDMS (Krayden, Denver, CO, USA) was weighed and mixed at a 10:1 base-to-curing agent ratio, poured onto a silicon master, degassed in a vacuum desiccator for approximately 30 min, and baked at 80 °C for at least 1 hour. Devices were cut out of the silicon master, and holes were punched into the PDMS to create cell and media inlets and outlets. Microchannels and 25 × 75 mm #1 glass coverslips (Electron Microscopy Sciences, Hatfield, PA, USA) were washed with ethanol and reverse osmosis or MilliQ water, dried for 5 min at 80 °C, then plasma treated in plasma cleaner (Harrick Plasma, Ithaca, NY, USA) for 2.5 min. The devices were bonded to the glass coverslips by applying pressure for 3 to 5 min, then UV sterilized for 10 min. Finally, devices were coated with 20 μg/mL collagen I (Sigma Aldrich, St. Louis, MO, USA) for 1 hour at 37 °C. After incubation, collagen was removed, devices were washed twice with PBS, and cells were immediately seeded into the cell inlet.

During the collagen I coating incubation time period, cells were prepared for device seeding. Cells were removed from the incubator, washed once with PBS, and detached with the addition of trypsin or TrypLE for 5 min. FBS-containing media was then added, and the cells were centrifuged at 1000 RPM for 5 min. Cells were resuspended in FBS-free media, counted, centrifuged again, and resuspended again in FBS-free media to yield 100,000 cells per 25 μl. Twenty-five microliters of cell suspension was pipetted into the cell inlets of the devices and incubated. After 5 min, excess cell suspension was removed, 50 μl of FBS-free media was added to the cell inlet and bottom two media inlets, and 50 μl of FBS-containing media was added to the topmost inlet to create a chemoattractant gradient. For studies including blebbistatin (50 μM, Sigma Aldrich, St. Louis, MO, USA) or nocodazole (10 μM, Sigma Aldrich, St. Louis, MO, USA), the inhibitor was added to both FBS-free and FBS-containing media, such that it was in equal concentrations throughout the device. Dimethylsulfoxide (Sigma Aldrich, St. Louis, MO, USA) served as the vehicle control in all experiments. Cells were incubated overnight to allow for migration through the channels. All microchannel devices containing cells were cultured at 37 °C, 50% humidity, and 5% CO_2_: 95% air.

### 2.3. Immunofluorescence

Following an overnight incubation period, cells and media were removed from the inlets and outlets. In all future steps listed below, when a reagent was added, it is implied that the same volume was added to all wells. Cells were washed once with PBS and immediately fixed with 3.7% formaldehyde (Fisher Scientific, Fair Lawn, NJ, USA). After a 10-min incubation at room temperature, cells were washed thrice with PBS. Cells were permeabilized by the addition of 0.5% Triton-X 100 (Sigma Aldrich, St. Louis, MO, USA) for 5 min, followed by three PBS washes. Nonspecific binding was then blocked with 2.5% goat serum (Abcam, Cambridge, MA, USA) for 1 hour at room temperature. Primary antibody diluted in PBS and 1% goat serum was added, and samples were incubated overnight at 4 °C. Primary antibody used was mouse anti-α-tubulin (ThermoFisher Scientific, Waltham, MA, USA, #A11126, 1:100 or 1:200). The next day, cells were washed twice or thrice in PBS, incubated in 2.5% goat serum for at least 1 hour and incubated with AlexaFluor 488 Phalloidin (ThermoFisher Scientific, Waltham, MA, USA, 1:500), Hoechst (ThermoFisher Scientific, Waltham, MA, USA, 1:500 or 1:2500), and a fluorescently labeled secondary antibody for 1 hour. Secondary antibody used was AlexaFluor 568 goat anti-mouse (ThermoFisher Scientific, Waltham, MA, USA, #A11004, 1:200). Cells were washed twice or thrice in PBS and imaged immediately or within a few days when Fluoromount-G (ThermoFisher Scientific, Waltham, MA, USA) was added for preservation.

### 2.4. Imaging

Widefield or confocal microscopy was used to extrapolate nuclear dimensions. MSC, MDA-MB-231, and L929 images for 2D measurement were acquired on an Olympus IX83 microscope (Olympus, Tokyo, Japan) using a 40× or 60× oil immersion objective. All z-stack images were taken using a PerkinElmer UltraVIEW Vox confocal spinning disk microscope (PerkinElmer, Waltham, MA) with a 100x oil immersion objective. Z-step size was 0.1 or 0.2 μm, and the boundaries of the stack were defined as the planes in which no staining could be observed. Use of the PerkinElmer confocal microscope was performed courtesy of the University of Maryland imaging core. In all experiments, fluorescence intensity and sensitivity were adjusted manually to optimize visualization.

### 2.5. Data Analysis in 2D

All data analysis for calculating 2D nuclear major axis length, minor axis length, and area was completed in ImageJ (U. S. National Institutes of Health, Bethesda, Maryland, USA) using the built-in morphology measurements. Widefield images were used for analysis. Nuclei were manually traced and measured, then dimensions originally output as pixels were converted into microns for analysis. Specifically, to calculate nuclear major and minor axis lengths, ImageJ fitted the traced nuclei to ellipses.

### 2.6. Data Analysis in 3D

ImageJ was used to quantify volume from the acquired z-stacks. First, the edges of the z-stacks were cut according to the built-in ImageJ feature ‘Find Edges’. This was completed to minimize the effects of background fluorescence during thresholding. The top and bottom planes were defined as the first and last image plane, respectively, in which an edge of the nucleus was visible. Next, IsoData thresholding was performed by ImageJ. IsoData threshold can be expressed mathematically as $Threshold=\;\frac{average\;background+average\;objects}{2}$. This automated thresholding method was chosen to exclude any bias in the analysis, and for its ability to threshold consistently for nuclei in all channel widths. Thresholding was calculated based on the middle plane of the stack and applied to all other planes in the stack. After thresholding, the ImageJ feature ‘Fill Holes’was used to correct any large holes in the interior of the nuclei caused by excess thresholding or low fluorescence signal. The ImageJ feature ‘3D Object Counter’ was then used to automatically calculate the volume based on the number of voxels of the object. 3D Object Counter also yielded the length, width, and height of the object bounding box. This methodology was validated by seeding 10-μm, yellow-green fluorescent polystyrene microspheres (ThermoFisher Scientific, Waltham, MA, USA) inside the channels and completing a similar imaging protocol to experimental conditions. In a 50-μm wide channel and a z-step size of 0.15 μm, the volume calculated of the microsphere with the described methodology was 570 μm^3^, in comparison with the expected volume of 524 μm^3^, which yields an error percentage of 8.8%. 

### 2.7. Statistical Analysis

**Figure 1:**
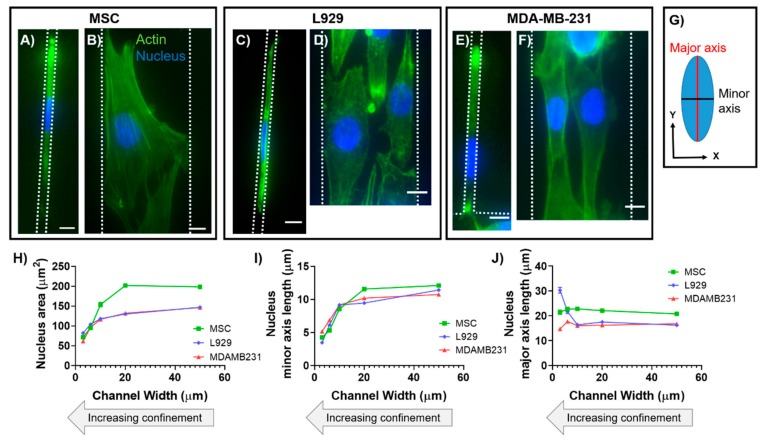
Quantification of 2D nuclear morphology in confinement. Images are shown for mesenchymal stem cells (MSCs) within microchannel widths of (**A**) 3 μm and **B**) 50 μm. Images are shown for L929 cells within microchannel widths of (**C**) 3 μm and (**D**) 50 μm. Images are shown for MDA-MB-231 cells within microchannel widths of (**E**) 3 μm and (**F**) 50 μm. In panels A–F cells were fixed and stained for actin (green) and the nucleus (blue). Color channels were altered individually for optimal visualization. Scale bar represents 10 μm in panels A–F. (**G**) Definition of the nuclear major and minor axes. Also shown are quantifications of the nucleus (**H**) area, (**I**) minor axis, and (**J**) major axis of MSCs, MDA-MB-231 cells, and L929 cells. Markers on line graphs report mean ± SEM of n cells, pooled from N ≥ 2 independent experiments with *n*(3 μm, MSC) = 9; *n*(3 μm, MDA-MB-231) = 178; *n*(3 μm, L929) = 26, *n*(6 μm, MSC) = 25; *n*(6 μm, MDA-MB-231) = 114; *n*(6 μm, L929) = 21; *n*(10 μm, MSC) = 21; *n*(10 μm, MDA-MB-231) = 98; *n*(10 μm, L929) = 80; *n*(20 μm, MSC) = 104; *n*(20 μm, MDA-MB-231) = 288; *n*(20 μm, L929) = 44; *n*(50 μm, MSC) = 289; *n*(50 μm, MDA-MB-231) = 620; *n*(50 μm, L929) = 136. Full statistical information for panels H–J is provided in Supplemental Tables S1–S3.

To compare nuclear dimensions in different channel widths, a one-way ANOVA with Holm-Sidak’s multiple comparisons test, in the case of normally distributed data, or Kruskal–Wallis test with a Dunn’s post hoc test, in the case of non-normally distributed data, was completed. Data were pooled from at least three independent trials, except in 2D data for MSC and MDA-MB-231, which were pooled from two independent trials. A significance level of 0.05 was used. Error bars report the standard error of the mean. Select graphs were unable to fit all comparisons, in which case we refer readers to the Figure 1H and supplementary tables for full statistical analysis.

## 3. Results

### 3.1. Nuclear Deformation as a Function of Confinement is Cell Type-Dependent

We have previously shown that MSC morphology and cytoskeletal arrangement is altered with increasing confinement [13], and here we aimed to determine whether cells from different species origins, tissue locations, and disease states displayed similar behaviors. We chose to examine MSCs, MDA-MB-231 cells, and L929 cells. These cell types were chosen due to their differences in tissue origin (bone marrow, mammary gland, and subcutaneous connective tissue, respectively), organism origin (human, human, and mouse, respectively), and disease pathology (healthy, cancerous, and healthy, respectively). Furthermore, these cells are extensively used throughout literature in many different applications, and therefore we found them to be relevant cell models. We first visualized the nuclear morphology of the three cell types within microchannels of varying width (Figure 1). We note that 50 μm wide channels served as our ‘unconfined’ control, where cells were not constrained in the XY plane. Indeed, cell nuclei in 3-μm narrow channels consistently appeared quite elongated and ellipsoidal in the XY plane (Figure 1A,C,E). As the channels widened, the nuclei became more circular in the XY plane as the cells themselves became more spread (Figure 1B,D,F). We first quantified whether there were differences in XY nucleus area, major axis length, or minor axis length (Figure 1G) with increasing confinement (i.e., decreasing channel width). In all cell types, nucleus area decreased with increasing confinement, albeit with different levels of significance (Figure 1H and Supplemental Table S1). Similarly, the nucleus minor axis decreased with increasing confinement for all cell types (Figure 1I and Supplemental Table S2). Note that due to the manual tracing of widefield microscopy images, some minor axis values were slightly above their respective microchannel width. We maintain that this variability was consistent across all channel widths. Interestingly, cell type-dependent trends emerged for nucleus major axis length. MSCs and MDA-MB-231 cells maintained a fairly constant nucleus major axis length as a function of channel width, with some exceptions (Figure 1J and Supplemental Table S3). L929 cells showed a marked increase in nucleus major axis length with increasing confinement (Figure 1J and Supplemental Table S3). These trends in L929 cell nuclei were similar to what we have previously viewed in mouse sarcoma cells [14]. In summary, the nucleus major axis deformed differently in response to confinement for MSCs and MDA-MB-231 cells than for L929 cells (and our previously published mouse sarcoma cells). Having made these 2D observations in the XY plane, we next investigated if there were cell type-dependent differences in nucleus height and volume during deformation in confinement.

### 3.2. Nuclear Deformation in 3D is Cell Type Dependent

**Figure 2:**
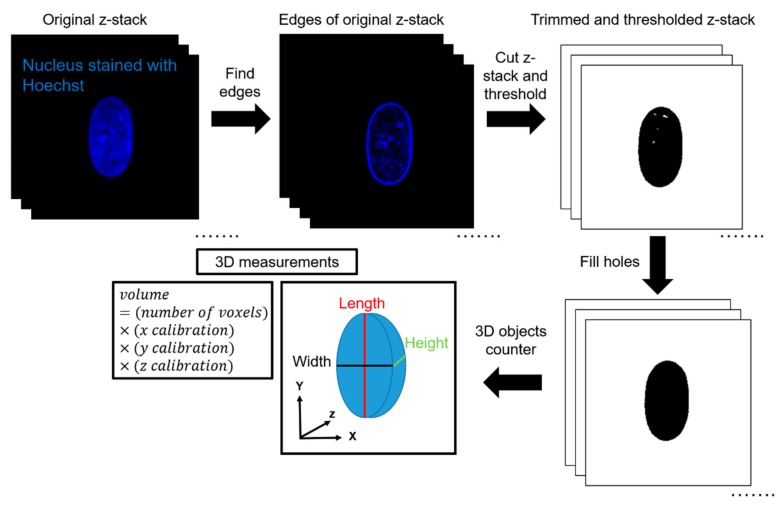
Workflow for calculating 3D morphological parameters of the nucleus.

**Figure 3:**
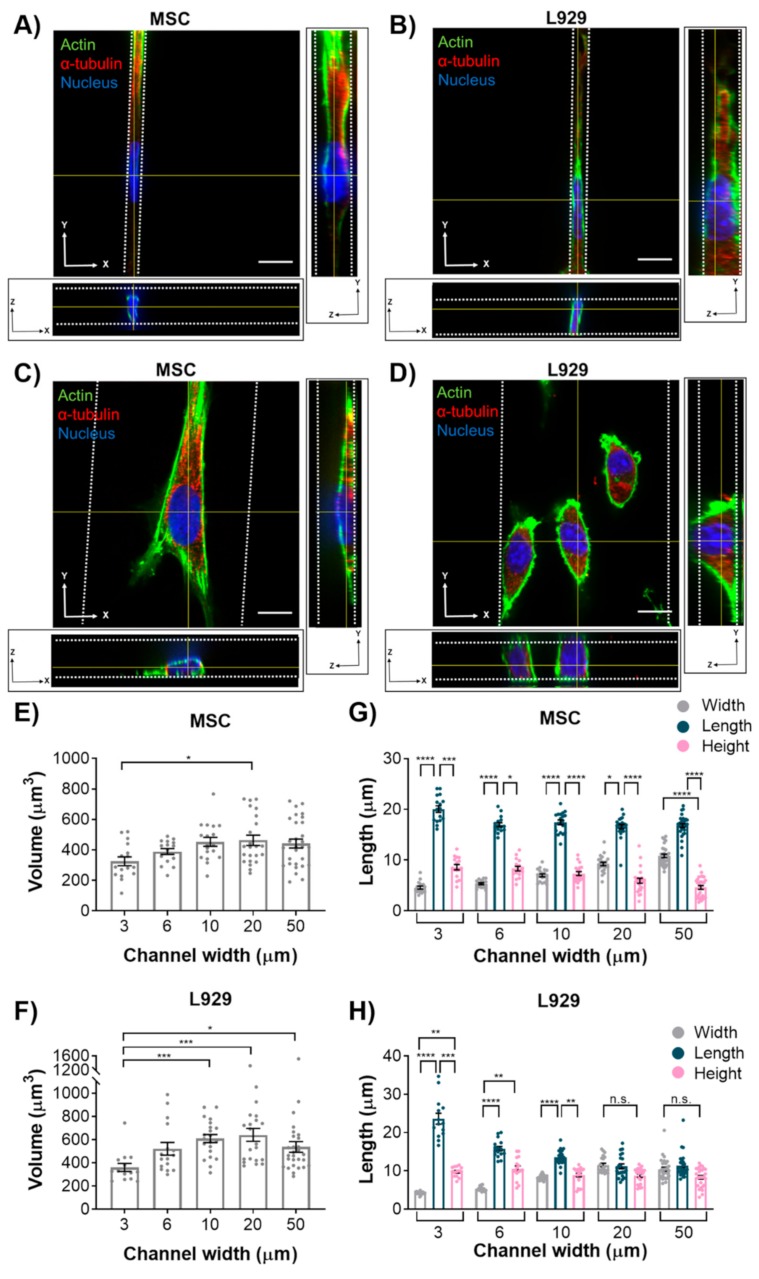
Orthogonal views of (**A**) MSC and (**B**) L929 cell within 3-μm narrow channel. Orthogonal views of (**C**) MSC and (**D**) L929 cell within 50-μm wide channel. In panels A–D cells were fixed and stained for α-tubulin (red), actin (green), and the nucleus (blue). Color channels were altered individually for optimal visualization. Scale bar represents 10 μm and applies to panels A–D. 3D renderings of nuclei shown in panels A–D are provided in Supplemental videos S1–4. Also shown are quantifications of the nucleus volume of (**E**) MSCs and (**F**) L929 cells, along with nucleus length, width, and height of (**G**) MSCs and (**H**) L929 cells. Dot plots report mean ± SEM. * *p* < 0.05, ** *p* < 0.005, *** *p* < 0.0005, **** *p* < 0.0001. (Each dot indicates one cell, pooled from N ≥ 3 independent experiments.) Full statistical information for panels G–H is provided inSupplemental Tables S4–S7.

To investigate the effects of confinement on nuclear height and volume, we narrowed our experimental cell lines to two. We selected MSCs and L929 cells due to their opposing trends in major axis length with increasing confinement, as measured from 2D images. Using confocal microscopy, we captured z-stacks of cells within each microchannel width. Using ImageJ, we quantified nuclear volume and the length, width, and height of the nucleus (Figure 2). Due to a high level of background fluorescence, we calculated the nuclear volume of a shortened z-stack, as indicated by the Find Edges tool in ImageJ. However, the images herein show the full z-stack. Therefore, a high amount of background fluorescence is evident above the confining microchannel in some figures. Both MSCs and L929 cells in 3-μm narrow channels appeared elongated, with diffuse microtubule structures and a defined actin ring around the cell perimeter (Figure 3A,B and Supplementary Videos S1 and S2). As the channels widened, more actin stress fibers formed within MSCs, and the MSC nucleus became flattened in the z-axis (Figure 3C and Supplementary Video S3). In contrast, L929 cell nuclei maintained a similar height in the z-axis as channels widened, and their cytoskeletal structures remained more diffuse than MSCs (Figure 3D and Supplementary Video S4). Length was comparable to the major axis in 2D, and width was comparable to the minor axis in 2D. Nuclear volume in 20-μm wide channels was significantly increased in both MSC and L929 cells when compared to 3-μm wide channels (Figure 3E,F). Additionally, L929 nuclei within 10- or 50-μm wide channels were significantly larger in volume than nuclei within 3-μm wide channels (Figure 3F). In MSCs, nuclei in 3- and 6-μm channels were significantly smaller in width than MSCs in 50-μm channels, but nuclear length and height were unchanged with decreasing channel width (Figure 3G and Supplementary Tables S4 and S5). Similarly, L929 cell nuclei in 3-, 6-, and 10-μm wide channels all had significantly shorter widths than cell nuclei within 20-μm channels (Figure 3H and Supplementary Tables S6 and S7). In contrast with MSCs, the L929 nuclear length in 3-μm wide channels was significantly longer than nuclei in 20- or 50-μm wide channels (Figure 3H and Supplementary Tables S6 and S7). Despite similar trends in nuclear volume, L929 cells and MSCs differed in nuclear dimensionality. MSCs remained rather anisotropic in all channel widths; MSC nuclear length was significantly longer than nuclear width and height in all channel widths (Figure 3G). L929 cell nuclei had similar anisotropic behavior in 3-, 6-, and 10-μm wide channels. However, L929 nuclei in 20- and 50-μm wide channels were isotropic, with no differences in nuclear length, width, or height (Figure 3H). Upon observing differences in nuclear volume and dimensionality with increasing confinement, we questioned if this behavior would be altered by perturbations to the cytoskeleton.

### 3.3. Microtubule Polymerization is not Necessary to Maintain Nuclear Morphology in Confinement

**Figure 4:**
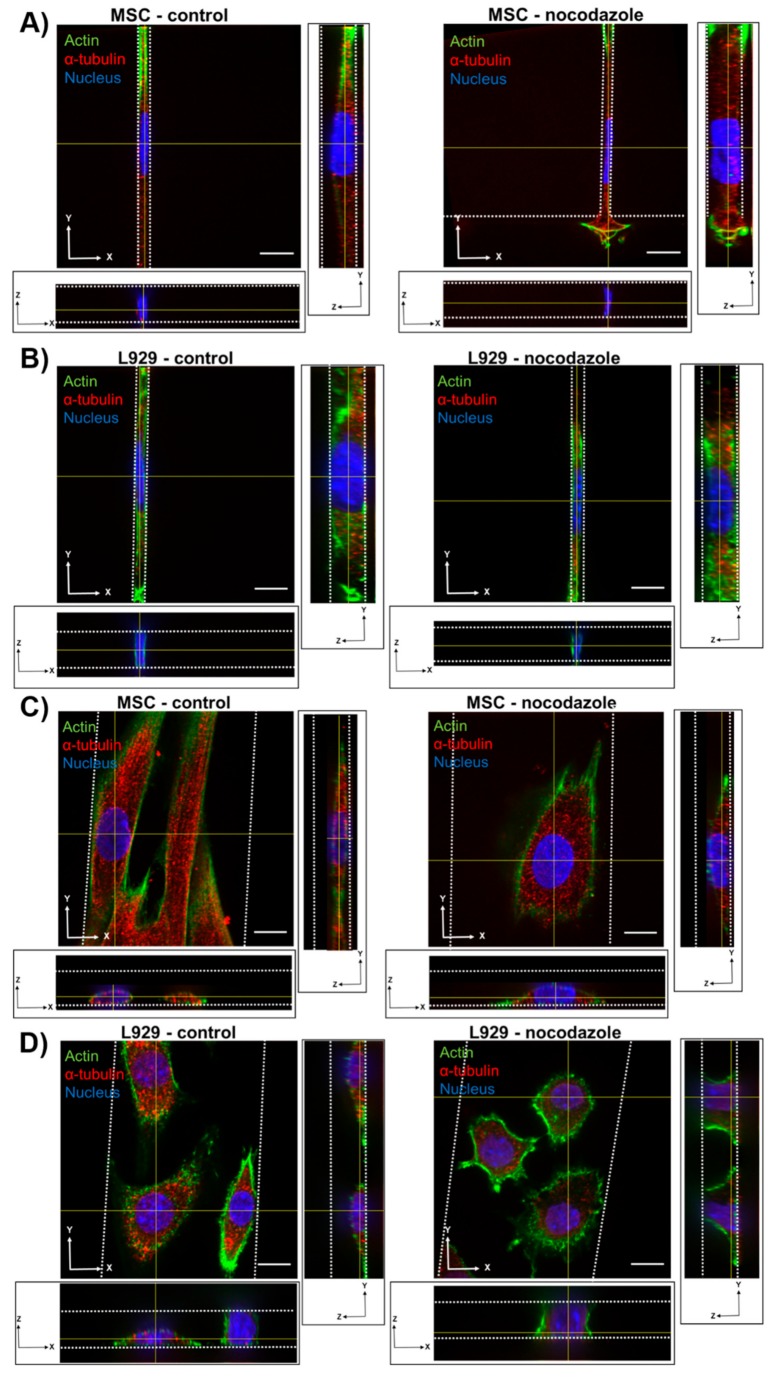
Orthogonal views of MSC treated with 10 μM nocodazole or vehicle control within a (**A**) 3-μm narrow channel or (**B**) 50-μm wide channel. Orthogonal views of L929 cell treated with 10 μM nocodazole or vehicle control within a (**C**) 3-μm narrow channel or (**D**) 50-μm wide channel. Cells were fixed and stained for α-tubulin (red), actin (green), and the nucleus (blue). Color channels were altered individually for optimal visualization. Scale bar represents 10 μm. 3D renderings of nuclei shown in panels A–D are provided in Supplemental Videos S5–S12.

**Figure 5:**
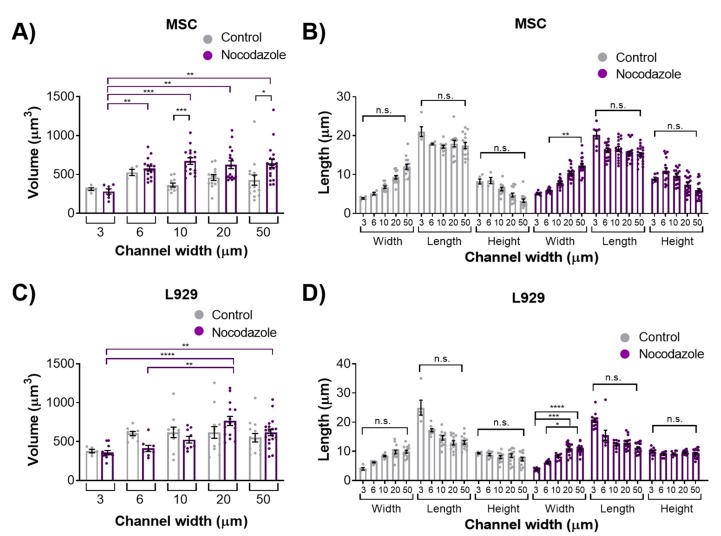
Nucleus (**A**) volume and (**B**) length, width, and height of MSCs treated with 10 μM nocodazole or vehicle control. Nucleus (**C**) volume and (**D**) length, width, and height of L929 cells treated with 10 μM nocodazole or vehicle control. Dot plots report mean ± SEM. * *p* < 0.05, ** *p* < 0.005, *** *p* < 0.0005, **** *p* < 0.0001. (Each dot indicates one cell, pooled from N ≥ 3 independent experiments.) Full statistical information for panels B and D is provided inSupplemental Tables S8–S11.

To investigate the role of the microtubule network in maintaining nuclear volume and dimensionality in confinement, we inhibited microtubule polymerization in MSCs and L929 cells by adding 10 μM nocodazole to cell media. Nocodazole-treated cells within 3-μm narrow channels appeared similar to the control, with diffuse cytoskeletal features in both cell types (Figure 4A,B and Supplementary Videos S5–S8). However, in 50-μm wide channels, nocodazole-treated MSCs and L929 cells appeared rounder, with less evidence of linear microtubule structures (Figure 4C,D and Supplementary Videos S9–S12). MSCs treated with nocodazole in 10- and 50-μm wide channels contained nuclei with significantly larger volumes than the control (Figure 5A). Although the nuclear heights appeared slightly larger in nocodazole-treated cells compared to the control, there was no significant difference in nuclear axis lengths between the nocodazole-treated and control groups for the same channel widths (Figure 5B and Supplementary Tables S8 and S9). L929 cells treated with nocodazole showed no difference in volume or nuclear axis lengths from the controls of the same channel width (Figure 5C,D and Supplementary Tables S10 and S11).

### 3.4. Myosin II Contractility is not Necessary to Maintain Nuclear Morphology in Confinement

**Figure 6:**
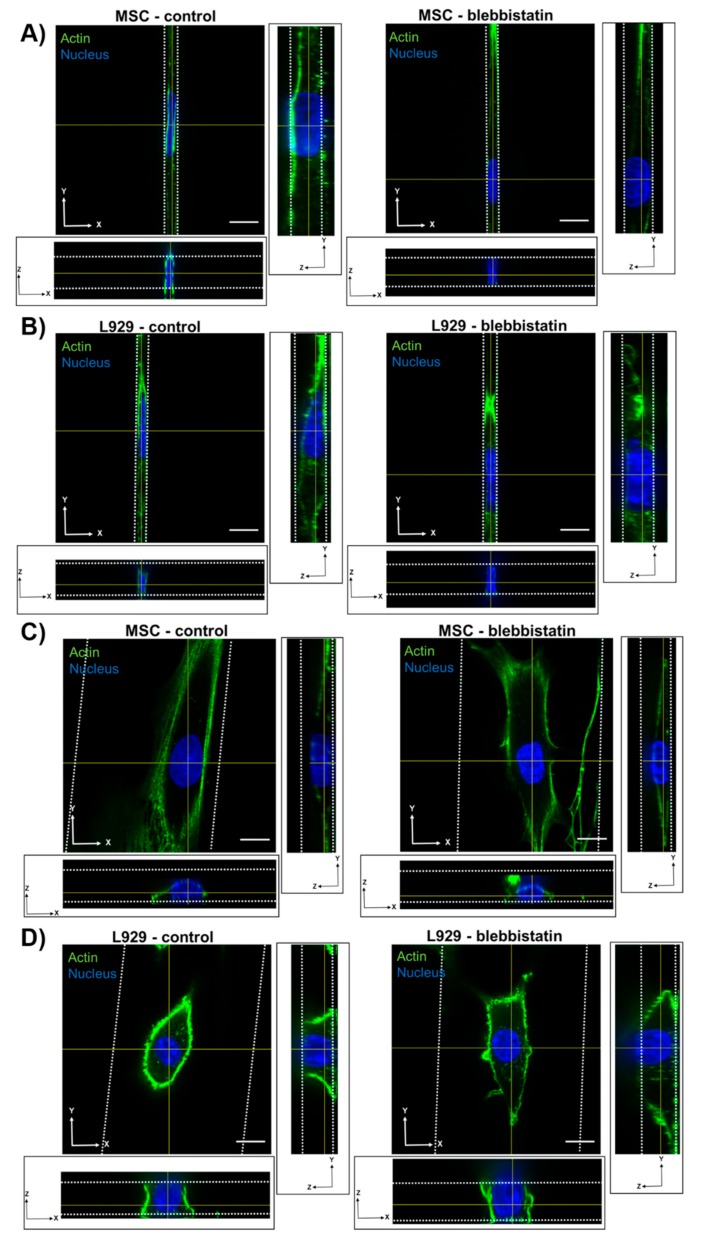
Orthogonal views of MSC treated with 50 μM blebbistatin or vehicle control within (**A**) 3-μm narrow channel and (**B**) 50-μm wide channel. Orthogonal views of L929 cell treated with 50 μM blebbistatin or vehicle control within (**C**) 3-μm narrow channel and (**D**) 50-μm wide channel. Cells were fixed and stained for actin (green) and the nucleus (blue). Color channels were altered individually for optimal visualization. Scale bar represents 10 μm. 3D renderings of nuclei shown in panels A–D are provided in Supplemental Videos S13–S20.

**Figure 7:**
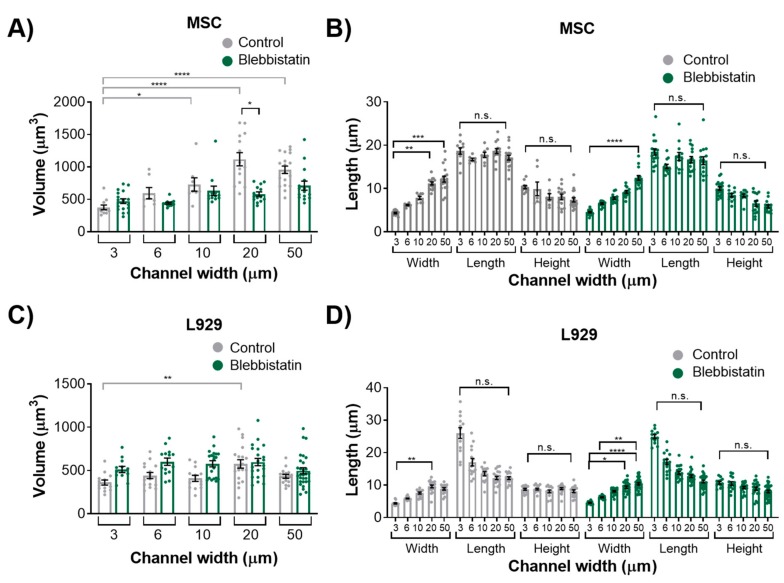
Nucleus (**A**) volume and (**B**) length, width, and height of MSCs treated with 50 μM blebbistatin or vehicle control. Nucleus (**C**) volume and (**D**) length, width, and height of L929 cells treated with 50 μM blebbistatin or vehicle control. Dot plots report mean ± SEM. * *p* < 0.05, ** *p* < 0.005, *** *p* < 0.0005, **** *p* < 0.0001. (Each dot indicates one cell, pooled from N ≥ 3 independent experiments.) Full statistical information for panels B and D is provided in Supplemental Tables S12–S15.

To investigate the role of the actomyosin network in maintaining nuclear volume and dimensionality in confinement, we inhibited myosin II-mediated contractility by adding 50 μM blebbistatin to cell media. In both wide and narrow microchannels, the actin organization did not appear drastically different between blebbistatin and control groups (Figure 6 and Supplementary Videos S13–S20). Some blebbistatin-treated L929 cells in wide channels exhibited a longer trailing edge than control cells (Figure 6D). We have previously shown that MSCs in microchannels do not exhibit altered microtubule structure upon blebbistatin treatment [13]. MSCs treated with blebbistatin in 20-μm wide channels displayed nuclei with significantly less volume than MSCs treated with vehicle control (Figure 7A). However, MSCs treated with blebbistatin did not show any differences in any axis lengths from the control (Figure 7B and Supplementary Tables S12 and S13). L929 cells treated with blebbistatin showed no difference in volume or nuclear axis lengths from control (Figure 7C,D and Supplementary Tables S14 and S15).

## 4. Discussion

In this study, we investigated how the passive force of confinement in microchannels affected nuclear deformation of various cell types. In 2D, nuclei of MSCs and MDA-MB-231 cells maintained a fairly constant major axis length when the minor axis was forcibly decreased by confining microchannel walls. In contrast, nuclei of L929 cells increased their major axis length as the minor axis was forcibly decreased by microchannels, which is similar to what we have previously shown in mouse sarcoma cells [14]. It has also been reported in literature that the nuclei of several cell lines deform anisotropically in response to an applied force, with the minor axis exhibiting higher strain than the major axis [8]. These results were demonstrated to be both intrinsic to the nucleus and controlled by the cytoskeleton, and it was postulated that microtubules resist nuclear deformation along the major axis, while actin resists deformation along the minor axis [8].

We initially hypothesized that nuclear volume would not change with increasing confinement, leading us to a second hypothesis that the height of the MSC nuclei would increase with increasing confinement, whereas the height of L929 nuclei would remain constant. However, interestingly, nuclei of MSCs and L929 cells decreased in volume when they experienced the highest degree of confinement. This result is consistent with other studies that have reported changes in nuclear volume in response to mechanical microenvironments. For example, nuclear volume was altered by nanotopography, potentially mediated by focal adhesion arrangement [31]. When confined to microwells, nuclear volume decreased with decreasing microwell volume [32]. Additionally, we observed a large range in the values of nucleus volume, which were on par with what others have reported [12,29]. When MSCs were confined to microwells in a previous study, their nuclear volume was less than observed here [32]; however, the microwells in that study were approximately 10 kPa in elastic modulus, whereas the microchannels herein were approximately 1 MPa in elastic modulus. This suggests that there may be a complex interplay between physical confinement and matrix stiffness in regulating nuclear volume.

MSC nuclei were anisotropic in all channel widths, while L929 cell nuclei were anisotropic in the narrowest channels and isotropic in the widest channels. MSCs exhibited more defined actin stress fibers than L929 cells in wide channels. Given the flattened morphology of MSCs in wide channels, we hypothesize that actin stress fibers compress the MSC nucleus in wide channels, thereby reducing nuclear height and increasing nuclear width with decreasing confinement. MSCs increased nuclear height in response to increasing confinement, as nuclear width was forced to decrease. Interestingly, the MSC nuclear length remained constant in all channel widths. Conversely, L929 cells maintained the same nuclear height in all channel widths. We suggest that this was due to minimal stress fiber formation and minimal cytoskeletal tension in L929 cells, which were more rounded. To compensate, L929 cells increased nuclear length in response to increasing confinement, as nuclear width was forced to decrease. It is interesting to note that L929 cells and MSCs have different origins—from mouse adipose and human bone marrow, respectively. It was previously observed that nuclear stiffness scales with tissue stiffness due to differences in lamin A content [28]. Although adipose and bone marrow are both soft tissues, MSCs have higher lamin A content than cells derived from fat or lung tissue [28]. Chromatin condensation may also affect nuclear deformation in confinement. It has been shown that chromatin decondensation leads to a more-isotropic deformation of fibroblast nuclei [8]. Additionally, it has been shown that polarized, elongated cells (similar to MSCs in wide channels) generate higher stress on the nucleus, have higher lamin A/C levels, and have less dynamic heterochromatin foci [33]. Conversely, isotropic cells contain a rounder nucleus, lower lamin A/C levels, and more dynamic heterochromatin foci [33]. These chromatin dynamics may ultimately influence nuclear deformation.

We observed the inhibition of microtubule polymerization via nocodazole increased MSC nuclear volume within wider 10- and 50-μm channels. Nocodazole has been shown to increase nuclear volume and increase major axis strain in 2D [8,29]. In two dimensions, microtubules seem to exert a force on the nucleus, as evidenced by a crescent-shaped nucleus around the microtubule organizing center (MTOC) when lamin A is knocked out [33], and by decreases in nuclear envelope fluctuations after nocodazole treatment [34]. Furthermore, inhibition of microtubule polymerization in the long term decreases elasticity of fibroblasts in 2D [35]. Similarly, we showed previously that nocodazole did not affect MSC migration speed in confinement, but reduced cell speed in wide channels [13]. It is possible that microtubule polymerization has a reduced role in regulating nuclear morphology in confinement compared with 2D microenvironments. Nocodazole did not, however, affect the nuclear volume or dimensions of L929 cells in any channel width. The microtubule network in L929 cells already appeared quite diffuse in all channel widths before treatment, suggesting that microtubules in these cells contribute little to maintaining nuclear morphology even in 2D microenvironments.

We also observed that inhibition of myosin II contractility decreased the nuclear volume of MSCs in 20-μm wide channels. Blebbistatin is often used to decrease the stiffness of cortical actin [36], and may have reduced the force on the nucleus exerted by the actin network. Inhibition of myosin II by blebbistatin has been shown to decrease the force on the nucleus and increase the levels of phosphorylated lamin A/C, which is typically associated with lamin A/C turnover and rounding nuclei [37]. Untreated L929 cells did not appear to have many actin stress fibers in any channel width, and hence they may have a low-contractile basal state. Therefore, the addition of blebbistatin did not seem to alter the cytoskeletal structure of L929 cells. In reporting these results, we also note an inherent limitation in our methods: Confocal microscopy is a costly and time-consuming technique, so it is difficult to obtain a significantly large sample size for the quantification of nuclear 3D volume and dimensions. Future studies could work towards developing a high-throughput method of confining and imaging cells, which could enable larger sample sizes to support our conclusions.

It does not appear that the external effectors of cell contractility or microtubule polymerization are key determinants of nuclear dimensions in confinement, which leaves the question of whether internal nuclear effectors control nuclear volume in confinement. Future studies may investigate the effects of chromatin decondensation or lamina disruption on nuclear volume, as these have both been demonstrated to decrease or increase, respectively, the anisotropic nucleus deformation in response to force [8]. Additionally, the LINC complex has been demonstrated to control the homeostatic position of the nucleus [38]. It is possible that the LINC complex may have a role in nuclear volume regulation or nuclear deformation in response to force. Internal nuclear effectors ought to be investigated for their role in nuclear deformation in three dimensions.

## 5. Conclusions

In summary, we observed that different cell types deform differently in response to mechanical confinement. Microtubule polymerization and myosin II contractility do not appear essential in L929 cells for regulating of nuclear volume and dimensionality. Conversely, microtubule polymerization and myosin II contractility seem to play a role in maintaining nuclear volume and dimensionality in unconfined, but not confined, MSCs. This work informs our understanding of nuclear mechanics in microenvironments related to tissue homeostasis and disease, and suggests diverging roles of the cytoskeleton in regulating nuclear deformation in different cell types.

## Supplemental Material

The following are available online at https://www.mdpi.com/2073-4409/8/5/427/s1, Tables S1–S15: statistical comparisons for Figure 1, Figure 3, Figure 5 and Figure 7, Videos S1–S20: 3D renderings of cell nuclei.
