# Supplementary material for: Nuclear Deformation in Response to Mechanical Confinement is Cell Type Dependent
Source: Cells. 2019 May 8;8(5):427. doi: 10.3390/cells8050427 (PMC6563141; doi:10.3390/cells8050427)
Supplement: Supplementary file 1 [file cells-08-00427-s001.zip › mdoolin-cells-supplemental.pdf]

Supplementary Material for

Nuclear Deformation in Response to Mechanical Confinement is Cell Type Dependent

*Mary T. Doolin[1], *Thea S. Ornstein[1], Kimberly M. Stroka[1,2,3,4]

[1]Fischell Department of Bioengineering, University of Maryland, College Park, MD, 20742, USA

[2]Biophysics Program, University of Maryland, College Park, MD, 20742, USA

[3]Center for Stem Cell Biology and Regenerative Medicine, University of Maryland – Baltimore, MD, 21201, USA

[4]Marlene and Stewart Greenebaum Comprehensive Cancer Center, University of Maryland – Baltimore, MD, 21201, USA

Table S1. Statistics for nucleus area for MSCs, L929 cells, and MDA-MB-231 cells, ns=not significant, *p<0.05, **p<0.005, ****p<0.0001.

Area	MSC	L929	MDAMB231
3 vs 6 µm	ns	ns	****
3 vs 10 µm	ns	****	****
3 vs 20 µm	****	****	****
3 vs 50 µm	****	****	****
6 vs 10 µm	ns	ns	**
6 vs 20 µm	****	*	****
6 vs 50 µm	****	****	****
10 vs 20 µm	****	ns	*
10 vs 50 µm	****	****	****
20 vs 50 µm	ns	ns	ns

Table S2. Statistics for nucleus minor axis for MSCs, L929 cells, and MDA-MB-231 cells, ns=not significant, *p<0.05, **p<0.005, ***p<0.0005, ****p<0.0001.

Minor axis	MSC	L929	MDAMB231
3 vs 6 µm	ns	ns	**
3 vs 10 µm	****	****	****
3 vs 20 µm	****	****	****
3 vs 50 µm	****	****	****
6 vs 10 µm	****	***	****
6 vs 20 µm	****	****	****
6 vs 50 µm	****	****	****
10 vs 20 µm	****	ns	**
10 vs 50 µm	****	****	****
20 vs 50 µm	*	****	ns

Table S3. Statistics for nucleus major axis for MSCs, L929 cells, and MDA-MB-231 cells, ns=not significant, *p<0.05, **p<0.005, ***p<0.0005, ****p<0.0001.

Major axis	MSC	L929	MDAMB231
3 vs 6 µm	ns	ns	****
3 vs 10 µm	ns	****	ns
3 vs 20 µm	ns	****	ns
3 vs 50 µm	ns	****	***
6 vs 10 µm	ns	****	***
6 vs 20 µm	ns	*	****
6 vs 50 µm	*	****	***
10 vs 20 µm	ns	ns	ns
10 vs 50 µm	**	ns	ns
20 vs 50 µm	***	ns	ns

Table S4. Statistics for MSC nucleus dimensions ns = not significant, **p<0.005, ****p<0.0001.

	Width	Length	Height
3 vs 6 µm	ns	ns	ns
3 vs 10 µm	ns	ns	ns
3 vs 20 µm	**	ns	ns
3 vs 50 µm	****	ns	ns
6 vs 10 µm	ns	ns	ns
6 vs 20 µm	ns	ns	ns
6 vs 50 µm	**	ns	ns
10 vs 20 µm	ns	ns	ns
10 vs 50 µm	ns	ns	ns
20 vs 50 µm	ns	ns	ns

Table S5. Statistics for MSC nucleus dimensions ns = not significant, *p<0.05, **p<0.005, ***p<0.0005, ****p<0.0001.

	Channel width (µm)				
	3	6	10	20	50
Width vs. length	****	****	****	*	ns
Width vs. height	ns	ns	ns	ns	****
Length vs. height	***	*	****	****	****

Table S6. Statistics for L929 nucleus dimensions ns = not significant, *p<0.05, **p<0.005, ***p<0.0005, ****p<0.0001.

	Width	Length	Height
3 vs 6 µm	ns	ns	ns
3 vs 10 µm	ns	ns	ns
3 vs 20 µm	****	**	ns
3 vs 50 µm	****	**	ns
6 vs 10 µm	ns	ns	ns
6 vs 20 µm	****	ns	ns
6 vs 50 µm	***	ns	ns
10 vs 20 µm	*	ns	ns
10 vs 50 µm	ns	ns	ns
20 vs 50 µm	ns	ns	ns

Table S7. Statistics for L929 nucleus dimensions ns = not significant, *p<0.05, **p<0.005, ***p<0.0005, ****p<0.0001.

	Channel width (µm)				
	3	6	10	20	50
Width vs. length	****	****	****	ns	ns
Width vs. height	**	**	ns	ns	ns
Length vs. height	***	ns	**	ns	ns

Table S8. Statistics for MSC nucleus dimensions when treated with 10 µM nocodazole or vehicle control ns = not significant, **p<0.005, ***p<0.0005, ****p<0.0001.

		Channel width (µm)				
Comparison		3	6	10	20	50
Control	W vs. L	***	ns	***	ns	ns
	W vs. H	ns	ns	ns	ns	***
	L vs. H	ns	ns	***	****	****
Nocodazole	W vs. L	****	****	**	ns	ns
	W vs. H	ns	ns	ns	ns	**
	L vs. H	ns	ns	ns	**	****
W	Cont. vs. Noc.	ns	ns	ns	ns	ns
L	Cont. vs. Noc.	ns	ns	ns	ns	ns
H	Cont. vs. Noc.	ns	ns	ns	ns	ns

Table S9. Statistics for MSC nucleus dimensions when treated with 10 μM nocodazole or vehicle control ns = not significant, **p<0.005.

		Dimension		
Comparison		W	L	H
Control	3 vs. 6 μm	ns	ns	ns
	3 vs. 10 μm	ns	ns	ns
	3 vs. 20 μm	ns	ns	ns
	3 vs. 50 μm	ns	ns	ns
	6 vs. 10 μm	ns	ns	ns
	6 vs. 20 μm	ns	ns	ns
	6 vs. 50 μm	ns	ns	ns
	10 vs. 20 μm	ns	ns	ns
	10 vs. 50 μm	ns	ns	ns
	20 vs. 50 μm	ns	ns	ns
Nocodazole	3 vs. 6 μm	ns	ns	ns
	3 vs. 10 μm	ns	ns	ns
	3 vs. 20 μm	ns	ns	ns
	3 vs. 50 μm	ns	ns	ns
	6 vs. 10 μm	ns	ns	ns
	6 vs. 20 μm	ns	ns	ns
	6 vs. 50 μm	**	ns	ns
	10 vs. 20 μm	ns	ns	ns
	10 vs. 50 μm	ns	ns	ns
	20 vs. 50 μm	ns	ns	ns

Table S10. Statistics for L929 cell nucleus dimensions when treated with 10 μM nocodazole or vehicle control ns = not significant, *p<0.05, **p<0.005, ***p<0.0005, ****p<0.0001.

		Channel width (μm)				
Comparison		3	6	10	20	50
Control	W vs. L	****	****	**	ns	ns
	W vs. H	ns	ns	ns	ns	ns
	L vs. H	ns	**	**	ns	***
Nocodazole	W vs. L	****	***	ns	ns	ns
	W vs. H	ns	ns	ns	ns	ns
	L vs. H	*	ns	ns	ns	ns
W	Cont. vs. Noc.	ns	ns	ns	ns	ns
L	Cont. vs. Noc.	ns	ns	ns	ns	ns
H	Cont. vs. Noc.	ns	ns	ns	ns	ns

Table S11. Statistics for L929 cell nucleus dimensions when treated with 10 μM nocodazole or vehicle control ns = not significant, *p<0.05, ***p<0.0005, ****p<0.0001.

		Dimension		
Comparison		W	L	H
Control	3 vs. 6 μm	ns	ns	ns
	3 vs. 10 μm	ns	ns	ns
	3 vs. 20 μm	ns	ns	ns
	3 vs. 50 μm	ns	ns	ns
	6 vs. 10 μm	ns	ns	ns
	6 vs. 20 μm	ns	ns	ns
	6 vs. 50 μm	ns	ns	ns
	10 vs. 20 μm	ns	ns	ns
	10 vs. 50 μm	ns	ns	ns
	20 vs. 50 μm	ns	ns	ns
Nocodazole	3 vs. 6 μm	ns	ns	ns
	3 vs. 10 μm	ns	ns	ns
	3 vs. 20 μm	***	ns	ns
	3 vs. 50 μm	****	ns	ns
	6 vs. 10 μm	ns	ns	ns
	6 vs. 20 μm	ns	ns	ns
	6 vs. 50 μm	*	ns	ns
	10 vs. 20 μm	ns	ns	ns
	10 vs. 50 μm	ns	ns	ns
	20 vs. 50 μm	ns	ns	ns

Table S12. Statistics for MSC nucleus dimensions when treated with 50 μM blebbistatin or vehicle control ns = not significant, *p<0.05, **p<0.005, ***p<0.0005, ****p<0.0001.

		Channel width (μm)				
Comparison		3	6	10	20	50
Control	W vs. L	****	*	ns	ns	ns
	W vs. H	ns	ns	ns	ns	ns
	L vs. H	ns	ns	ns	***	****
Blebbistatin	W vs. L	****	**	**	ns	ns
	W vs. H	**	ns	ns	ns	**
	L vs. H	*	ns	*	****	****
W	Cont. vs. Bleb.	ns	ns	ns	ns	ns
L	Cont. vs. Bleb.	ns	ns	ns	ns	ns
H	Cont. vs. Bleb.	ns	ns	ns	ns	ns

Table S13. Statistics for MSC nucleus dimensions when treated with 50 μM blebbistatin or vehicle control ns = not significant, **p<0.005, ***p<0.0005, ****p<0.0001.

Comparison		Dimension		
		W	L	H
Control	3 vs. 6 μm	ns	ns	ns
	3 vs. 10 μm	ns	ns	ns
	3 vs. 20 μm	**	ns	ns
	3 vs. 50 μm	***	ns	ns
	6 vs. 10 μm	ns	ns	ns
	6 vs. 20 μm	ns	ns	ns
	6 vs. 50 μm	ns	ns	ns
	10 vs. 20 μm	ns	ns	ns
	10 vs. 50 μm	ns	ns	ns
	20 vs. 50 μm	ns	ns	ns
Blebbistatin	3 vs. 6 μm	ns	ns	ns
	3 vs. 10 μm	ns	ns	ns
	3 vs. 20 μm	ns	ns	ns
	3 vs. 50 μm	****	ns	ns
	6 vs. 10 μm	ns	ns	ns
	6 vs. 20 μm	ns	ns	ns
	6 vs. 50 μm	ns	ns	ns
	10 vs. 20 μm	ns	ns	ns
	10 vs. 50 μm	ns	ns	ns
	20 vs. 50 μm	ns	ns	ns

Table S14. Statistics for L929 cell nucleus dimensions when treated with 50 μM blebbistatin or vehicle control ns = not significant, *p<0.05, ***p<0.0005, ****p<0.0001.

Comparison		Channel width (μm)				
		3	6	10	20	50
Control	W vs. L	****	****	***	ns	ns
	W vs. H	ns	ns	ns	ns	ns
	L vs. H	****	***	*	ns	*
Blebbistatin	W vs. L	****	****	****	ns	ns
	W vs. H	***	*	ns	ns	ns
	L vs. H	ns	ns	*	*	*
W	Cont. vs. Bleb.	ns	ns	ns	ns	ns
L	Cont. vs. Bleb.	ns	ns	ns	ns	ns
H	Cont. vs. Bleb.	ns	ns	ns	ns	ns

Table S15. Statistics for L929 cell nucleus dimensions when treated with 50 μM blebbistatin or vehicle control ns = not significant, *p<0.05, **p<0.005, ****p<0.0001.

		Dimension		
Comparison		W	L	H
Control	3 vs. 6 μm	ns	ns	ns
	3 vs. 10 μm	ns	ns	ns
	3 vs. 20 μm	**	ns	ns
	3 vs. 50 μm	ns	ns	ns
	6 vs. 10 μm	ns	ns	ns
	6 vs. 20 μm	ns	ns	ns
	6 vs. 50 μm	ns	ns	ns
	10 vs. 20 μm	ns	ns	ns
	10 vs. 50 μm	ns	ns	ns
	20 vs. 50 μm	ns	ns	ns
Blebbistatin	3 vs. 6 μm	ns	ns	ns
	3 vs. 10 μm	ns	ns	ns
	3 vs. 20 μm	*	ns	ns
	3 vs. 50 μm	****	ns	ns
	6 vs. 10 μm	ns	ns	ns
	6 vs. 20 μm	ns	ns	ns
	6 vs. 50 μm	**	ns	ns
	10 vs. 20 μm	ns	ns	ns
	10 vs. 50 μm	ns	ns	ns
	20 vs. 50 μm	ns	ns	ns

Supplemental Movies: For all movies, the first frame represents the x-y plane, and the nucleus (stained with Hoechst) revolves around the y-axis. Stacks were thresholded and cut according to the workflow described in Figure 2 before being rendered in ImageJ. All scale bars on movies are in μm.

Movie S1. 3D rendering of MSC nucleus in 3 μm channel.
Movie S2. 3D rendering of L929 nucleus in 3 μm channel.
Movie S3. 3D rendering of MSC nucleus in 50 μm channel.
Movie S4. 3D rendering of L929 nucleus in 50 μm channel (center cell).

Movie S5. 3D rendering of MSC nucleus in 3 μm channel treated with vehicle control.
Movie S6. 3D rendering of MSC nucleus in 3 μm channel treated with nocodazole.
Movie S7. 3D rendering of L929 nucleus in 3 μm channel treated with vehicle control.
Movie S8. 3D rendering of L929 nucleus in 3 μm channel treated with nocodazole.
Movie S9. 3D rendering of MSC nucleus in 50 μm channel treated with vehicle control.
Movie S10. 3D rendering of MSC nucleus in 50 μm channel treated with nocodazole.
Movie S11. 3D rendering of L929 nucleus in 50 μm channel treated with vehicle control (bottom right cell).
Movie S12. 3D rendering of L929 nucleus in 50 μm channel treated with nocodazole (top cell).

Movie S13. 3D rendering of MSC nucleus in 3 μm channel treated with vehicle control.
Movie S14. 3D rendering of MSC nucleus in 3 μm channel treated with blebbistatin.
Movie S15. 3D rendering of L929 nucleus in 3 μm channel treated with vehicle control.
Movie S16. 3D rendering of L929 nucleus in 3 μm channel treated with blebbistatin.

Movie S17. 3D rendering of MSC nucleus in 50 μm channel treated with vehicle control.
Movie S18. 3D rendering of MSC nucleus in 50 μm channel treated with blebbistatin.
Movie S19. 3D rendering of L929 nucleus in 50 μm channel treated with vehicle control.
Movie S20. 3D rendering of L929 nucleus in 50 μm channel treated with blebbistatin.
